# Antimicrobial Resistance in *Enterococcus* Spp. Isolated from a Beef Processing Plant and Retail Ground Beef

**DOI:** 10.1128/Spectrum.01980-21

**Published:** 2021-11-17

**Authors:** Devin B. Holman, Cassidy L. Klima, Katherine E. Gzyl, Rahat Zaheer, Cara Service, Tineke H. Jones, Tim A. McAllister

**Affiliations:** a Lacombe Research and Development Centre, Agriculture and Agri-Food Canada, Lacombe, Alberta, Canada; b Lethbridge Research and Development Centre, Agriculture and Agri-Food Canada, Lethbridge, Alberta, Canada; Vetsuisse Faculty University of Zurich

**Keywords:** *Enterococcus*, beef, antimicrobial resistance, abattoir, multidrug resistance

## Abstract

Antimicrobial use in food-producing animals has come under increasing scrutiny due to its potential association with antimicrobial resistance (AMR). Monitoring of AMR in indicator microorganisms such as *Enterococcus* spp. in meat production facilities and retail meat products can provide important information on the dynamics and prevalence of AMR in these environments. In this study, swabs or samples were obtained from various locations in a commercial beef packing operation (*n* = 600) and from retail ground beef (*n* = 60) over a 19-month period. All samples/swabs were enriched for *Enterococcus* spp., and suspected enterococci isolates were identified using species-specific PCR primers. Enterococcus faecalis was the most frequently isolated species, followed by Enterococcus hirae, which was found mostly on post-hide removal carcasses and in ground beef. Enterococcus faecium (*n* = 9) and E. faecalis (*n* = 120) isolates were further characterized for AMR. Twenty-one unique AMR profiles were identified, with 90% of isolates resistant to at least two antimicrobials and two that were resistant to nine antimicrobials. Tetracycline resistance was observed most often in E. faecalis (28.8%) and was likely mediated by *tet*(M). Genomic analysis of selected E. faecalis and E. faecium isolates revealed that many of the isolates in this study clustered with other publicly available genomes from ground beef, suggesting that these strains are well adapted to the beef processing environment.

**IMPORTANCE** Antimicrobial resistance (AMR) is a serious challenge facing the agricultural industry. Understanding the flow of antimicrobial-resistant bacteria through the beef fabrication process and into ground beef is an important step in identifying intervention points for reducing AMR. In this study, we used enterococci as indicator bacteria for monitoring AMR in a commercial beef packaging facility and in retail ground beef over a 19-month period. Although washing of carcasses post-hide removal reduced the isolation frequency of *Enterococcus* spp., a number of antimicrobial-resistant Enterococcus faecalis isolates were recovered from ground beef produced in the packaging plant. Genome analysis showed that several E. faecalis isolates were genetically similar to publicly available isolates recovered from retail ground beef in the United States.

## INTRODUCTION

*Enterococcus* spp. are often used as indicators of fecal contamination due to their association with the mammalian gastrointestinal tract and persistence in the environment (1). The concentration of enterococci in the feces of cattle varies but is typically around 10^4^ to 10^5^ CFU g^−1^ (2, 3), and microbial contamination of beef carcasses can happen during hide removal and evisceration in beef processing facilities (4). Previous studies have reported that *Enterococcus* spp. are prevalent in ground beef samples in North America (5–8), but less information is available regarding the prevalence of enterococci in beef processing environments.

Presently, there are more than 60 species of *Enterococcus* and two subspecies (LPSN; http://www.bacterio.net), with Enterococcus faecalis and Enterococcus faecium associated most frequently with ground beef (5, 6). These species are considered commensal microorganisms in humans; however, certain E. faecalis and E. faecium strains are responsible for serious nosocomial infections and vancomycin-resistant enterococci (VRE) strains are particularly difficult to treat (9, 10) due to limited antimicrobial treatment options. Many enterococci are intrinsically resistant to several antimicrobials and can also acquire resistance through horizontal gene transfer and point mutations (11, 12).

Feedlots in North America have traditionally administered antimicrobials to cattle to prevent and treat disease (13). This includes classes of antimicrobials that are also used in human medicine, such as β-lactams, fluoroquinolones, macrolides, and tetracyclines (14, 15). There is concern that the use of antimicrobials in food-producing animals selects for antimicrobial-resistant bacteria that may be disseminated to humans through consumption of food and the environment (16). Additionally, antimicrobial-resistant strains of E. faecium isolated from meat have transiently colonized the human gastrointestinal tract when consumed in challenge experiments (17), and transfer of the tetracycline resistance gene, *tet*(M), from an E. faecium strain of meat origin to human clinical enterococci isolates has been demonstrated *in vitro* (18). The culturability and ubiquity of *Enterococcus* spp. in cattle make them ideal for monitoring antimicrobial resistance (AMR) in beef processing facilities and retail products.

Therefore, in this study we isolated enterococci from samples taken from a commercial beef processing facility over a 19-month period and from retail ground beef in Alberta. The objective was to determine the prevalence of enterococci on pre- and postwashed carcasses, on the conveyor belt area transporting beef cuts, and in ground beef produced within the beef plant and to characterize AMR in E. faecalis and E. faecium isolates recovered from these samples. We also wanted to assess how related certain E. faecalis and E. faecium isolates from this study were to each other and to a selection of publicly available E. faecalis and E. faecium genomes from various sources.

## RESULTS

### *Enterococcus* species distribution and prevalence.

Ten different *Enterococcus* species were isolated from swabs and ground beef samples, with E. faecalis, Enterococcus hirae, and E. faecium recovered most frequently (Table 1). Within the beef processing facility, the carcasses after hide removal and the ground beef yielded the greatest number of samples positive for enterococci. E. faecalis was the only species from all five sampling locations. The number of positive samples collected during the 15 different visits to the processing facility varied as well (Table S1). In 6 samples (out of 660), more than one *Enterococcus* sp. was identified (data not shown). Overall, enterococci were recovered from 39.0% of all samples from the facility using nonselective media, but on three separate sampling dates less than 20% of samples were positive. Only 14.2% of beef plant samples were positive for enterococci when grown on Enterococcosel agar supplemented with 8 μg erythromycin mL^−1^ (Table S1). Among these isolates from the selective media, *E*. *hirae* was predominant.

**TABLE 1:** Distribution and prevalence of *Enterococcus* spp. in swabs and samples from four different locations in a beef processing facility (*n* = 150) and in retail ground beef (*n* = 60); values represent the number of positive swabs or samples from nonselective media, and numbers in parentheses indicate the number of positive samples from selective (erythromycin) media

Species	No. positive swabs/samples from nonselective media (no. from selective media):				
	After hide removal	After final washing	Conveyor belt	Ground beef from processing facility	Ground beef from retail
Enterococcus faecalis	31 (1)	11	11	117 (2)	42 (1)
Enterococcus hirae	40 (38)	0 (3)	0	1 (30)	7 (10)
Enterococcus faecium	2 (2)	1 (1)	0	0 (5)	5 (2)
Enterococcus raffinosus	0	0	1	0 (1)	0
Enterococcus malodoratus	2	2	2	0	0
Enterococcus durans	5 (2)	0	0	0	0
Enterococcus gallinarum	0 (1)	0	0	0	1
Enterococcus casseliflavus	3	0	0	0	0
Enterococcus avium	0	0	0	0	1

The frequency of detection of enterococci on antibiotic-free Enterococcosel agar was similar for ground beef from the processing facility and that from retail locations (*P* > 0.05). Postwashed carcasses and the conveyor belt also did not differ in detection frequency (*P* > 0.05). The proportion of post-hide removal carcass samples positive for *Enterococcus* spp. was significantly higher than that of the postwashed carcasses and conveyor belt samples positive for *Enterococcus* spp. but also significantly lower than that of ground beef from the processing facility and retail locations positive for *Enterococcus* spp. (*P* < 0.05). However, on media supplemented with erythromycin, the frequency of enterococci isolation was similar among the ground beef samples and post-hide removal carcass swabs (*P* < 0.05). Recovery of enterococci from the postwash carcasses and conveyor belt was significantly less frequent on antibiotic-selective media than recovery of those from the other three sample types (*P* < 0.05) (Table S1).

### Antimicrobial susceptibility and detection of antimicrobial resistance genes.

Antimicrobial susceptibility testing was done on 120 E. faecalis and 9 E. faecium isolates using 16 different antimicrobials (Table S2). These isolates were randomly chosen to ensure that all location/sample types and sampling dates were covered and included isolates from Enterococcosel agar supplemented with erythromycin, as well. Nearly all E. faecalis isolates (erythromycin-supplemented and erythromycin-free media) were resistant to lincomycin (97.4%) and quinupristin-dalfopristin (93.2%) (Table 2; Table S2). Phenotypic resistance to ciprofloxacin (11.1%), erythromycin (12.8%), tetracycline (31.6%), and tylosin (6.8%) was also noted in several E. faecalis isolates. Although there were fewer E. faecium isolates available for testing, the AMR phenotypes were similar to E. faecalis with the exception of ciprofloxacin resistance, which was not observed in any of the E. faecium strains (Table S2). Two E. faecalis isolates (H11 and H22) from the carcasses after hide removal were resistant to nine antimicrobials, and one (G69E) from ground beef was resistant to six. Only one *Enterococcus* isolate was susceptible to all 16 antimicrobials tested, with no resistance recorded for linezolid, penicillin, or vancomycin in any of the isolates.

**TABLE 2:** Antimicrobial susceptibility for E. faecalis (*n* = 111) isolated on nonselective media by antimicrobial and isolation source^*a*^

Antimicrobial class	Percentage of resistant isolates (total no. of isolates):						
	Antimicrobial^*b*^	After hide removal (H)	After final washing (W)	Conveyor belt (C)	Ground beef from processing facility (G)	Ground beef from retail (R)	Total
Aminoglycosides	GEN	11.1% (2)	0	0	0	0	1.8% (2)
	KAN	11.1% (2)	0	0	0	0	1.8% (2)
	STR	11.1% (2)	0	0	0	0	1.8% (2)
Fluoroquinolones	CIP	5.6% (1)	0	28.6% (2)	11.8% (4)	11.6% (5)	10.8% (12)
Lincosamides	LIN	100% (18)	100% (9)	100% (7)	94.1% (32)	97.7% (42)	97.3% (108)
Lipopeptides	DAP	0	0	0	5.9% (2)	0	1.8% (2)
Macrolides	ERY	11.1% (2)	11.1% (1)	0	14.7% (5)	4.6% (2)	9.0% (10)
	TYL	11.1% (2)	0	0	2.9% (1)	2.3% (1)	3.6% (4)
Phenicols	CHL	11.1% (2)	0	0	0	0	1.8% (2)
Streptogramins	SYN	94.4% (17)	77.7% (7)	100% (7)	94.1% (32)	93.0% (40)	92.8% (103)
Tetracyclines	TET	11.1% (2)	11.1% (1)	14.3% (1)	50.0% (17)	25.6% (11)	28.8% (32)

a Values represent percentage of isolates that are resistant and numbers in parentheses indicate total number of isolates. None of the isolates were resistant to linezolid, nitrofurantoin, penicillin, tigecycline, or vancomycin.

b CHL, chloramphenicol; CIP, ciprofloxacin; DAP, daptomycin; ERY, erythromycin; GEN, gentamicin; KAN, kanamycin; LIN, lincomycin; STR, streptomycin; SYN, quinupristin-dalfopristin; TET, tetracycline; TYL, tylosin.

Among the 119 E. faecalis and 9 E. faecium isolates displaying phenotypic resistance to at least one antimicrobial, there were 21 unique AMR profiles (Table S3). The most common AMR profiles included resistance to quinupristin-dalfopristin and lincomycin (52.3%; 67) and quinupristin-dalfopristin, lincomycin, and tetracycline (20.3%; 26). The E. faecalis and E. faecium isolates were also screened for the presence of *erm*(B), *msrC*, *tet*(B), *tet*(C), *tet*(L), *tet*(M), *vanA*, *vanB*, and *vanC1* via PCR. The *tet*(M) (26.5%) and *erm*(B) (7.7%) genes were detected most frequently in E. faecalis and *msrC* (75.0%) and *erm*(B) (16.7%) in E. faecium. None of the *van* genes or *tet*(C) were found among these isolates. Of those E. faecalis isolates with phenotypic resistance to either erythromycin or tylosin, 47% carried the *erm*(B) gene, and the *tet*(L) or *tet*(M) genes were detected in 89% of those resistant to tetracycline (Table S2). In only one E. faecalis isolate was *erm*(B), *tet*(L), or *tet*(M) detected without corresponding phenotypic resistance.

### Genome analysis.

Forty-seven E. faecalis and eight E. faecium isolates were selected for whole-genome sequencing based on their AMR profiles and sample origin. The assembly statistics for these sequenced genomes are reported in Holman et al. (19) and Table S4. The size of the E. faecalis and E. faecium genomes ranged from 2,647,103 to 3,246,301 bp and 2,507,908 to 2,761,265 bp, respectively.

### Antimicrobial resistance genes within genome assemblies.

We screened the E. faecalis and E. faecium assemblies for antimicrobial resistance genes (ARGs) using the CARD RGI (Comprehensive Antibiotic Resistance Database Resistance Gene Identifier) and identified 15 different ARGs conferring resistance to 8 different antimicrobial classes. Similar to the PCR-based screening of select ARGs, *tet*(M) (31.9%) and *erm*(B) (8.5%) were found most often within E. faecalis genomes (Table 3). The genes *efrA*, *efrB*, *emeA*, and *lsa*(A), which encode multidrug efflux pumps (20, 21), were identified in all E. faecalis genomes, as was *dfrE*, a dihydrofolate reductase gene conferring resistance to diaminopyrimidine. Although the *efrAB* and *emeA* genes have been reported to increase the MIC of ciprofloxacin in transformed Escherichia coli strains (21, 22), the MIC values in those studies were well below the 4 μg mL^−1^ MIC breakpoint for resistance. Therefore, it appears unlikely that these genes contribute to clinical resistance to ciprofloxacin or any of the other antimicrobials tested against E. faecalis.

**TABLE 3:** Antimicrobial resistance genes identified in sequenced Enterococcus faecalis (*n* = 47) and Enterococcus faecium (*n* = 8) genomes

Gene	Product	Target	Percentage (no. genomes):	
			E. faecalis	E. faecium
*aac(6′)-Ii*	Acetyltransferase	Aminoglycosides	0	100% (8)
*ant(*6*)-Ia*	Nucleotidyltransferase	Aminoglycosides	4.3% (2)	0
*ant(*9*)-Ia*	Nucleotidyltransferase	Aminoglycosides	0	12.5% (1)
*aph(3′)-IIIa*	Phosphotransferase	Aminoglycosides	4.3% (2)	0
*lnuG*	Nucleotidyltransferase	Lincosamides	2.1% (1)	0
*msrC*	ABC transporter	Macrolides	0	100% (8)
*erm*(A)	23S rRNA methyltransferase	Macrolides	0	12.5% (1)
*erm*(B)	23S rRNA methyltransferase	Macrolides	8.5% (4)	12.5% (1)
*optrA*	ABC transporter	Oxazolidinones	0	12.5% (1)
*lpsB*	Intrinsic peptidentibiotic-resistant LPS	Peptides	2.1% (1)	0
*catA8*	Chloramphenicol acetyltransferase	Phenicols	2.1% (1)	0
*lsa*(E)	ABC transporter	Multiple drugs	4.3% (2)	0
*sat4*	Acetyltransferase	Streptothricins	4.3% (2)	0
*tet*(45)	Efflux protein	Tetracyclines	2.1% (1)	12.5% (1)
*tet*(M)	Ribosomal protection protein	Tetracyclines	31.9% (15)	37.5% (3)

All sequenced E. faecium genomes carried the *aac(6′)-Ii* and *msrC* genes conferring resistance to aminoglycosides and macrolides-lincosamides-streptogramin B, respectively. The *efmA* gene, which encodes a multidrug efflux pump (23), was found in all but one of the E. faecium genomes. The *aac(6′)-Ii*, *efmA*, and *msrC* genes are considered to be intrinsic within E. faecium (11). One E. faecalis strain (H11) that had been isolated from a carcass after hide removal but prior to washing carried 9 additional ARGs: *aac(6′)-Ie-aph(2′')-Ia*, *aad(6)*, *ant(6)-Ia*, *aph(3′)-IIIa*, *catA8*, *erm*(B), *lsaE*, *sat4*, and *tet*(M). A different E. faecalis strain (H22) also from a carcass post-hide removal had six additional ARGs: *aad(6)*, *ant(6)-Ia*, *aph(3′)-IIIa*, *lsaE*, *sat4*, and *tet*(M). These two isolates were phenotypically resistant to nine different antimicrobials and had the same multilocus sequence typing (MLST) profile but were collected 3 months apart. The only other isolate with more than two additional ARGs, E. faecalis H96E, was also collected from carcasses after removal of the hide.

**FIG 1:**
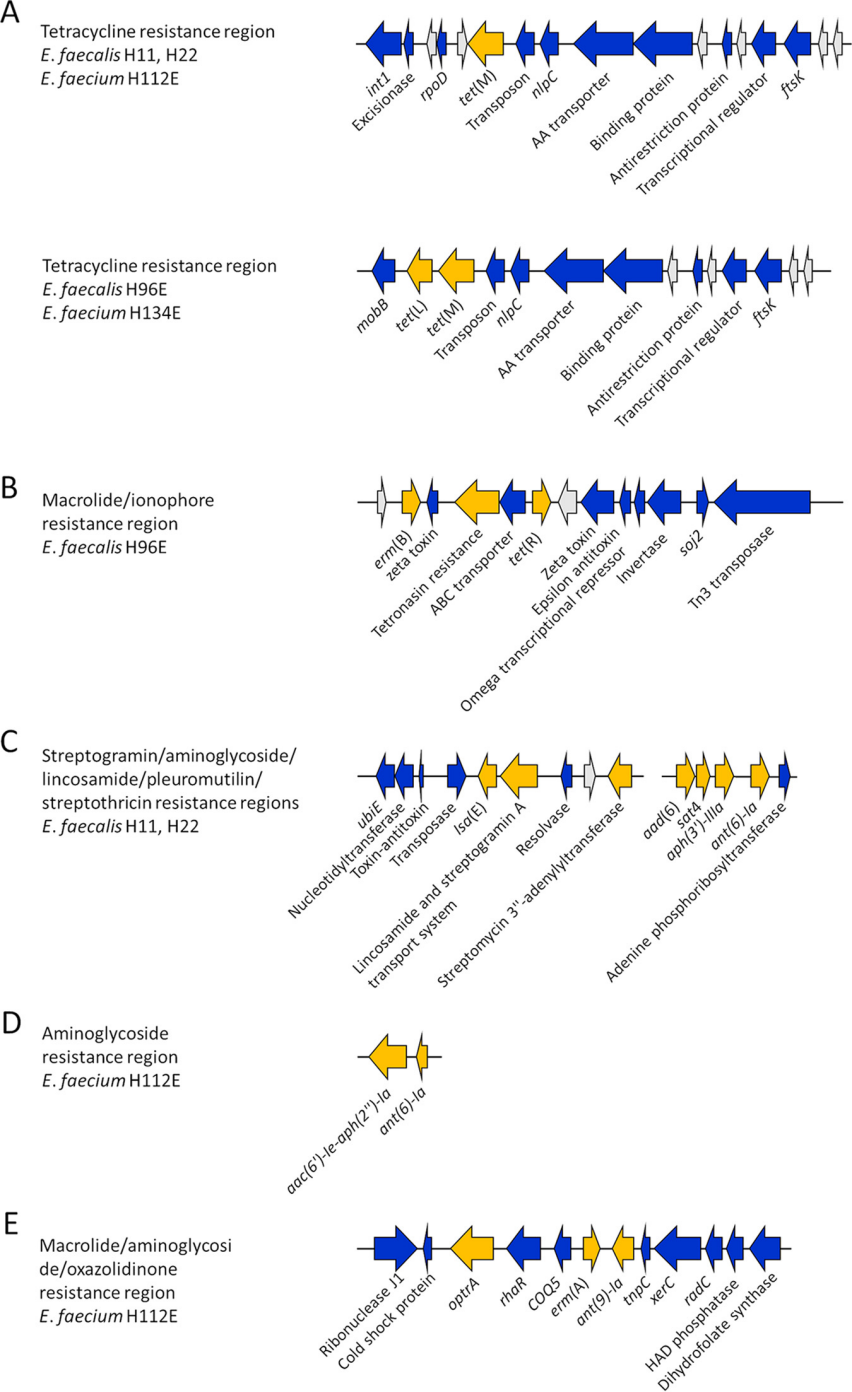
Location of antimicrobial resistance genes (ARGs) within indicated Enterococcus faecalis and Enterococcus faecium strains. The ARGs are displayed in yellow, non-ARGs genes are blue, and hypothetical proteins are colored gray.

Three E. faecalis (H11, H22, and H96E) and two E. faecium (H112E and H134E) isolates with multidrug resistance (presence of ARGs conferring resistance to three or more antimicrobial classes) profiles of interest were examined further to determine the genetic context of the ARGs detected. All five multidrug-resistant strains contained an insertion sequence harboring *tet*(M) (Fig. 1A) that had high sequence similarity (>80% identity and >70% coverage when aligned using E. faecium H134E) to integrative and conjugative elements found in Streptococcus suis (ICESsu05SC260, GenBank KX077888.1; ICESsuJH1308-2, GenBank KX077884.1). Alignment of this region in all five isolates showed 85% pairwise identity and revealed two variants with similarity in gene arrangements within E. faecalis H11, E. faecalis H22, and E. faecium H112E and between E. faecium H134E and E. faecalis H96E. Differences between the variants occurred on the left flank and included genes associated with integration and the presence of *tet*(L) [designated *tet*(45) by the CARD RGI] adjacent to *tet*(M) in H96E and H143E but not in H11, H22, and H112E. Despite complementarity, there were a significant number of point mutations in this region between H11, H22, and H112E (88% pairwise identify) that could reflect differences in the residence time of this gene region within each strain.

In E. faecalis H96E, a tetronasin resistance gene was identified approximately 60 kb upstream of *tet*(M), along with *erm*(B), a *tet*(R) gene, a transposase, a toxin-antitoxin system, and other genes associated with transcriptional regulation (Fig. 1B). The *erm*(B) gene was also present in E. faecalis H11 but was assembled as a single gene contig, and therefore its location within the genome could not be ascertained. The *lsa*(E) gene in E. faecalis H11 and H22 was found on contigs with identical gene arrangements that were truncated at the same location on the left and right flanks (Fig. 1C). In addition to *lsa*(E), these contigs also contained an unnamed streptomycin 3′-adenylyltransferase and a lincosamide and streptogramin A transport system ATP-binding/permease gene. The E. faecalis H11 and H22 assemblies also had contigs carrying the *aad(6)*, *sat4*, *aph(3′)-IIIa*, and *ant(6)-Ia* genes. Based on alignment against multiple *Enterococcus* strains in NCBI, the *sat4* gene-containing contig was adjacent on the chromosome to the contig carrying *lsa*(E), with the streptomycin 3′′-adenylyltransferase and *aad(6)* genes adjacent to each other. As with other ARG regions found in these isolates, strong pairwise identity was observed between parts of these contigs and similar cassettes found in Staphylococcus aureus strains (S. aureus BA01611, RefSeq NC_007795.1; S. aureus MRSA_S3, RefSeq NC_007795.1).

The aminoglycoside resistance genes *aac(6′)-Ie-aph(2″)-Ia* and *ant(6)-Ia* were found adjacent to one another, comprising a single contig in strain H11 (Fig. 1D). This couplet of ARGs is present in many E. faecium and E. faecalis strains in NCBI but can also be found in Staphylococcus spp., *Clostridium* spp., and Campylobacter coli strains. E. faecium H112E contained a gene region harboring the oxazolidinone resistance gene *optrA* in close proximity to the macrolide resistance gene *erm*(A), *ant(9)-Ia (*aminoglycoside resistance), and *xerC*, a tyrosine recombinase gene (Fig. 1E). This gene region aligned with complete coverage and greater than 99% identity to a plasmid in E. faecalis (GenBank CP042214.1) and an *optrA* gene cluster in E. faecium (GenBank MK251151.1), suggesting that this gene array could have originally been a plasmid that integrated into the chromosome of E. faecium H112E. Other ARGs present that assembled into single either gene contigs or gene regions lacking other ARGs were the lincosamide resistance gene *lunG* in E. faecalis H96E, the chloramphenicol resistance gene *catA*, and *msrC* in E. faecium H134E and H112E.

### Virulence genes.

Genome assemblies were also screened for virulence genes using the VirulenceFinder *Enterococcus* database. The virulence genes *ace* (collagen adhesin), *camE*, *cCF10*, *cOB1* (sex pheromones), *ebpA*, *ebpB*, *ebpC* (pili proteins), *efaAfs* (adhesion), *elrA* (enterococcal leucine rich protein A), *srtA* (sortase), and *tpx* (thiol peroxidase) were found in all E. faecalis genomes (Table S5). The gelatinase-encoding *gelE* and hyaluronidase genes *hylA* and *hylB* were also detected in 74.5%, 68.8%, and 83.0% of E. faecalis genomes, respectively. Only two E. faecalis genomes carried the cytolysin genes *cylABLM* and the extracellular surface protein (*eps*) gene, but notably, these were also the strains that had the greatest number of ARGs, H11 and H22. These genes were also detected only in the selected publicly available genomes that were isolated from humans. The *efaAfm* gene, which encodes a cell wall adhesin, was found in all eight E. faecium assemblies. The *acm* gene (collagen-binding protein) was the only other virulence gene detected in the E. faecium genomes (75%).

### Phylogeny of enterococcal strains.

Phylogenetic relationships among the 47 E. faecalis and 8 E. faecium strains from this study and 29 E. faecalis and 19 E. faecium genomes that were publicly available were determined using the core genes within each species. These additional E. faecalis and E. faecium genomes included all publicly available isolates from ground beef and several randomly selected human and cattle fecal isolates also from Alberta (24). The core genome of the 76 E. faecalis genomes contained 1,325 genes and the pan-genome had 9,558 genes. Among the 27 E. faecium genomes included for analysis, there were 1,417 genes in the core genome and 7,848 genes in the pan-genome.

**FIG 2:**
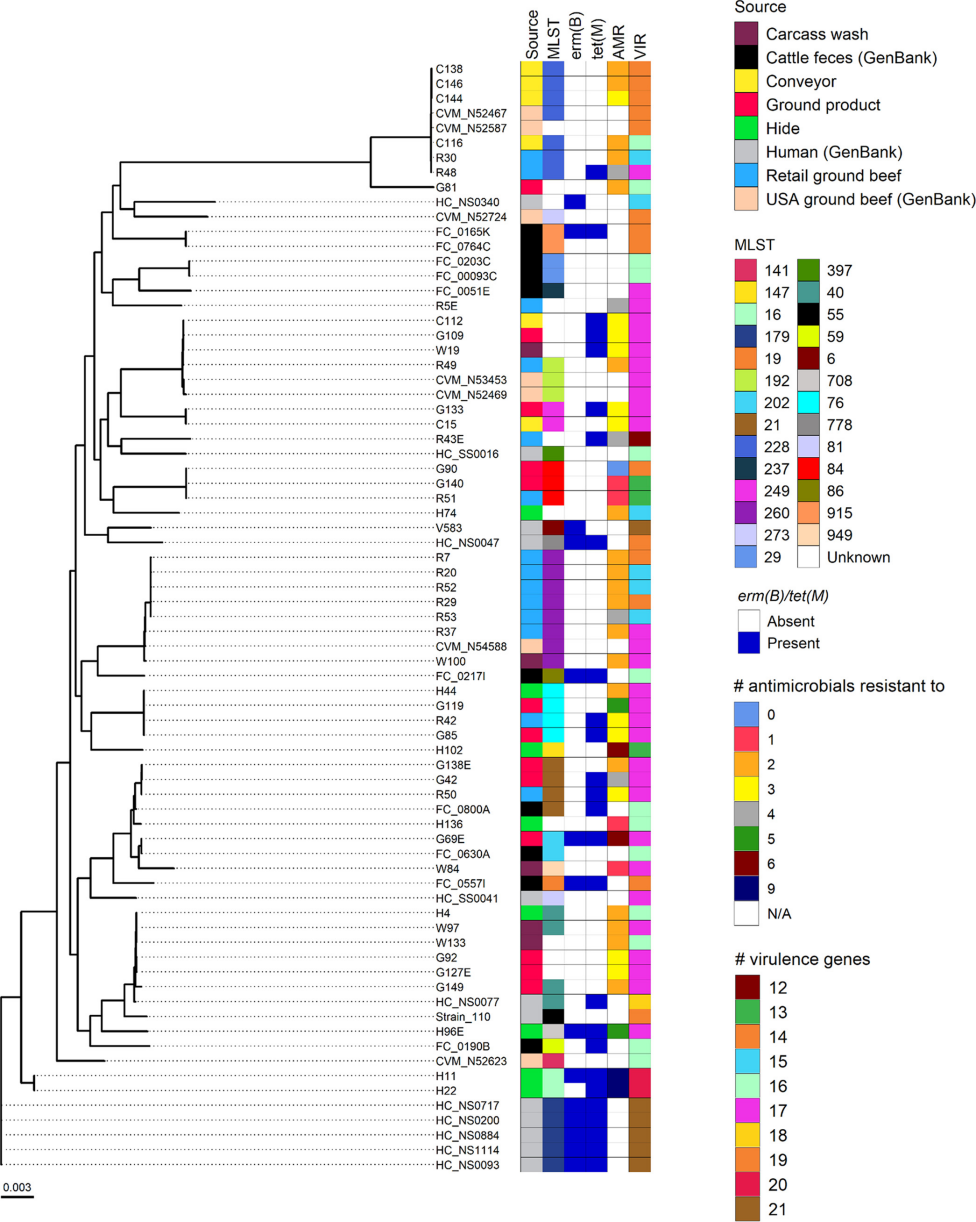
Maximum likelihood phylogeny of 47 Enterococcus faecalis isolates from the current study and selected publicly available E. faecalis genomes from cattle feces (*n* = 10), ground beef (*n* = 7), and humans (*n* = 12). Phylogeny was inferred from the alignment of 1,325 core genes using RAxML. Scale bar represents substitutions per nucleotide.

E. faecalis strains clustered by MLST type (Fig. 2). Among the 23 E. faecalis sequenced isolates from within the processing facility that could be assigned to a particular MLST profile, there were 12 unique MLST profiles. Interestingly, certain E. faecalis strains that had been collected from retail ground beef in the United States had an MLST profile (ST192, ST228, and ST260) that was shared with strains isolated from the conveyor belt, carcasses after final washing, and retail ground beef in the present study. Six of the E. faecalis isolates (G92, G127E, G149, H4, W97, and W133) had the same MLST profile as one of the Alberta human isolates (HC_NS0077). However, it should be noted that this human isolate carried *tet*(M) and an additional virulence gene which was absent in the six isolates from this study.

**FIG 3:**
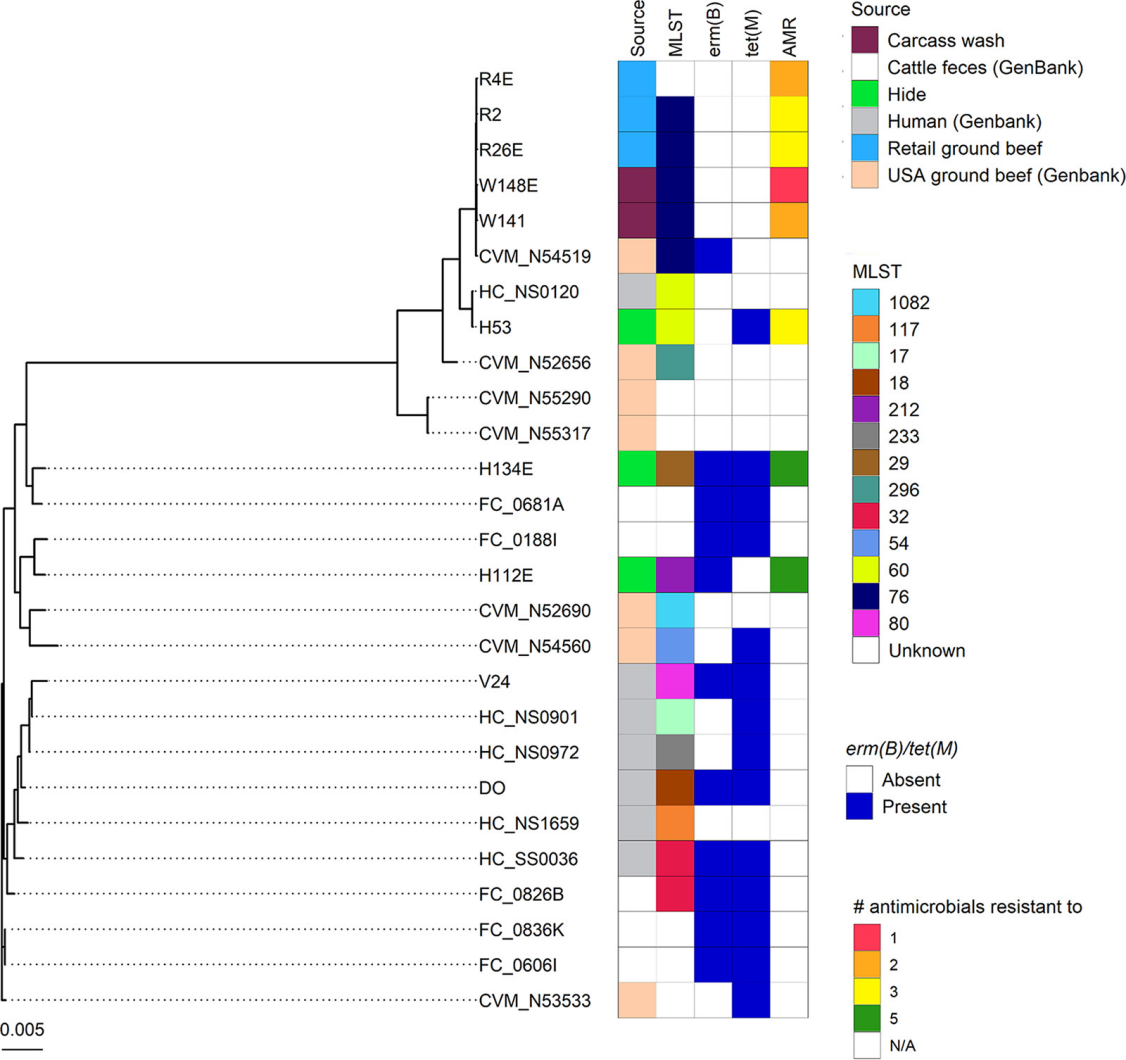
Maximum likelihood phylogeny of 8 Enterococcus faecium isolates and selected publicly available E. faecium genomes from cattle feces (*n* = 5), ground beef (*n* = 7), and humans (*n* = 7). Phylogeny was inferred from the alignment of 1,417 core genes using RAxML. Scale bar represents substitutions per nucleotide.

E. faecium isolates also clustered by MLST (Fig. 3). Three E. faecium isolates from retail ground beef along with two isolates from the postwash carcasses and one from U.S. ground beef had the same MLST (ST76). Unlike the E. faecalis genomes, there also appeared to be two distinct clades of E. faecium with the two post-hide removal isolates (H134E and H112E) in a separate clade from the other E. faecium isolates examined.

## DISCUSSION

AMR continues to be a serious public health threat, and there are concerns that antimicrobial-resistant bacteria in food-producing animals may be transferred to humans through the food production system. In this study, we used culturing and whole-genome sequencing to monitor AMR and enterococci distribution in a beef processing facility and in retail ground beef over a 19-month period. Although 10 different *Enterococcus* spp. were isolated at least once during the study, only E. faecalis was found in all sampling locations. This is consistent with previous surveys that sampled from beef plants (5) or retail ground beef (6). E. hirae was the species isolated most frequently from carcasses post-hide removal, which was expected given that *E*. *hirae* has been reported to be the most prevalent *Enterococcus* sp. in cattle feces (2, 24, 25) and there is greater likelihood of contamination from feces at the hide removal step (26). Notably, *E*. *hirae* was recovered more frequently from media supplemented with 8 μg erythromycin mL^−1^, likely in part due to the suppression of E. faecalis by erythromycin. Additionally, a study by Beukers et al. (2) reported that 42.9% of *E*. *hirae* isolates from cattle feces were resistant to erythromycin, as drugs of the macrolide class are frequently used to prevent and treat infectious disease in feedlot cattle.

The number of enterococcus-positive samples recovered from the carcasses postwashing and the conveyor belts was substantially lower than that from any other sample type. Carcasses are subjected to washing with hot water and spraying with organic acids after hide removal, which reduces the microbial load on the carcasses. The proportion of enterococci isolated from the conveyor belts was lower than that in an earlier study (10.7% versus 48%) (5). This may represent differences in sanitation or sampling methods within the conveyor area. However, 82.7% of the ground beef produced within the plant was positive for *Enterococcus* spp., most of which were E. faecalis, suggesting that the conveyor area is not a reflection of the prevalence of enterococci in the ground beef produced. The source of enterococci in the ground beef is unknown, but microbial contamination of ground beef can happen during the trimming and grinding processes from equipment surfaces, workers, and the environment. In the current study, this contamination may have occurred within either processing or retail environments. Enterococci were also isolated from the majority of ground beef samples taken from retail stores in Alberta, which was similar to previous surveys of enterococci in retail ground beef in Alberta (65%) (5) and the United States (92.7%) (6).

We subjected 120 of the E. faecalis and 9 of the E. faecium isolates to antimicrobial susceptibility testing, as these two species are opportunistic pathogens in humans. Of the antimicrobials classified by the World Health Organization (WHO) as critically important in human medicine (27), infrequent resistance to ciprofloxacin, daptomycin, erythromycin, gentamicin, kanamycin, and tigecycline was noted. None of the isolates were resistant to vancomycin or linezolid, which are antimicrobials often used to treat VRE strains (28). Resistance to lincomycin and quinupristin-dalfopristin is intrinsic in E. faecalis and mediated by the chromosomally carried *lsa*(A) gene (29), thus explaining the widespread resistance of E. faecalis to these antimicrobials. Tetracycline resistance was observed in 28.8% of E. faecalis isolates from erythromycin-free media and was likely mediated by the *tet*(M) gene, which encodes a ribosomal protection protein and was detected in 83.3% of tetracycline-resistant E. faecalis isolates and absent in tetracycline-susceptible ones. This finding is similar to previous reports of *tet*(M) in E. faecalis from beef and other foods (5, 30). Feedlot cattle in Western Canada have historically received tetracyclines such as chlortetracycline and oxytetracycline in feed or via injection for treatment and prevention of disease, possibly accounting for the prevalence of tetracycline resistance noted here (14).

Of the 47 E. faecalis genomes that were sequenced here, 31.9% also carried the *tet*(M) gene, as did 3 of the 8 E. faecium genomes sequenced. In *Enterococcus* spp., *tet*(M) is typically found within the Tn*916*-Tn*1545* family of conjugative transposons (31, 32). In this study, we examined the genetic context of the *tet*(M) gene and other ARGs in the isolates with phenotypic resistance to the greatest number of antimicrobials. In these isolates, *tet*(M) also appeared to be adjacent to transposases, as did *erm*(B) in E. faecalis strain H96E. Interestingly, the *erm*(B) gene in this particular isolate was found on the same contig as a tetronasin resistance gene. Tetronasin is an ionophore: a class of antimicrobials that is widely used in livestock production to prevent coccidiosis and promote growth (14). However, because ionophores are employed only in veterinary medicine, it is assumed that their use does not affect human health (33). To date, several studies have examined ionophore resistance in *Enterococcus* spp. but have reported little or no concern for its development (34). If any degree of resistance was observed, it was attributed to thickening of the cell wall or glycocalyx, traits that were considered to be genetically unstable and reversible upon removal of selective pressure (35).

An isolate from the current study was found to harbor the *erm*(B) gene near a tetronasin resistance gene. Linkages between ionophore resistance and ARGs from other drug classes are not unprecedented, with enterococci isolated from various locations around the world and from both humans and animals having been found to contain putative narasin resistance ABC transporters and *vanA* genes (33). It is important to note that *vanA* was not detected in any of the isolates in the present study and no isolates displayed phenotypic resistance to vancomycin. Furthermore, ionophore resistance was not phenotypically confirmed in this single isolate and further work would be required to determine if the use of ionophores could coselect for macrolide resistance in this strain. A large portion of the ARG cassettes examined here are also found in *Streptococcus*, *Staphylococcus*, and *Campylobacter* spp. in the NCBI nucleotide database. Future research that examines the rates of prevalence and transmissibility of these mobile regions between and among these species could be of considerable value in limiting the spread of AMR in bacteria of importance in human disease.

Several of the E. faecalis and E. faecium isolates from the postwashed carcasses, conveyor belt area, and ground beef from the plant and retail locations were genetically very similar to publicly available isolates from ground beef in the United States, suggesting that these particular strains are well adapted to the beef processing environment or possibly cattle. These may be strains that are transferred during beef processing or a result of cross-contamination of ground beef from equipment, workers, and/or the environment within the plant. The cytolysin and extracellular surface protein genes are virulence genes often associated with human clinical strains and increased toxicity (36, 37). Here, only two isolates, both from the carcasses after hide removal (E. faecalis H11 and H22), carried either of these genes, although these were also the strains that were resistant to the greatest number of antimicrobials. A low prevalence of these virulence genes in enterococci from retail ground beef in Alberta has also been reported previously (38). Of the 12 human-derived E. faecalis genomes included in this analysis, only one (HC_NS0077) appeared to be closely related to any of the E. faecalis isolates sequenced here. One E. faecium isolate from a carcass after hide removal was also genetically similar to a human E. faecium isolate (HC_NS0120), but this in itself does not constitute evidence of directional transfer.

In summary, longitudinal sampling from a commercial beef packaging facility revealed the presence of E. faecalis in all sample types (carcasses, conveyor belt, and ground beef), with the greatest prevalence found in ground beef produced in the plant. Whole-genome sequencing of selected E. faecalis and E. faecium isolates showed that certain isolates from different sample types were genetically very similar, suggesting a common origin, although that origin is unknown. Several multidrug-resistant isolates were recovered, including two E. faecalis isolates from carcasses post-hide removal which were resistant to nine different antimicrobials and carried a number of ARGs on potentially mobile elements. However, the risk that such strains found on the carcasses post-hide removal may pose to the food production system is unknown, as they were not isolated in the downstream processing environment.

## MATERIALS AND METHODS

### Sampling and isolation of *Enterococcus* spp.

Samples were collected a total of 15 times from July 2014 through February 2016 from a commercial beef processing facility in Alberta, Canada. During each visit, 10 samples were obtained from each of four different areas within the plant: carcasses after hide removal (H), carcasses after final washing and evisceration (W), conveyer belts (C), and the ground beef product (G). A 2 cm by 2 cm gauze swab was used to sample a randomly selected 10 cm by 10 cm area on the surface of the carcasses and conveyor belts. Conveyor belt swabs were taken while the conveyor was in use and transporting cuts of meat. In total, 150 samples were obtained from each sample type or location. During the same time period, 60 samples of retail ground beef (R) were collected from various retail locations in Alberta, which may or may not have arisen from the processing plant, as the origin of these retail ground beef samples was unknown. All samples were transported to the lab on ice and processed immediately. The swabs and 25 g of each ground product and retail ground beef sample were transferred to a stomacher bag for homogenization and preenrichment with 10 mL (swabs) or 225 mL (ground product/beef) of buffered peptone water. These samples were then stomached at 260 rpm for 2 min in a Stomacher 400 circulator (Seward, Norfolk, UK) and incubated overnight at 37°C.

One milliliter of this mixture was then added to 9 mL of Enterococcosel broth (BD, Mississauga, ON, Canada) with and without 8 μg erythromycin mL^−1^ (Sigma-Aldrich Canada, Oakville, ON, USA) and incubated overnight at 37°C for the enrichment of enterococci. Erythromycin was chosen since macrolides are important in human and veterinary medicine and enterococci are not intrinsically resistant to this antimicrobial. Enterococcosel broth tubes displaying evidence of esculin hydrolysis (black) were streaked onto Enterococcosel agar (BD) with and without 8 μg erythromycin mL^−1^ and incubated at 37°C. After 48 h, the plates were examined for colonies with black zones (esculin hydrolysis) and three colonies from each plate were restreaked onto Enterococcosel agar and incubated for 48 h at 37°C. Each positive colony was then transferred to 1 mL of brain heart infusion (BHI) broth (Dalynn Biologicals, Calgary, AB, Canada) containing 15% glycerol and stored at −80°C. Confirmation and species identification of presumptive enterococci isolates were done via PCR with the Ent-ES-211-233-F and Ent-EL-74-95-R primers (39) to amplify the *groES*-*EL* spacer region as described previously (2). Enterococcus hirae isolates were identified using primers mur2h-F 5′-TATGGATACACTCGAATATCTT-3′ and 5′-ATTATTCCATTCGATTAACTGC-3′ to target the muramidase (*mur-2*) gene of *E*. *hirae* as per Zaheer et al. (24). The *groES*-*EL* amplicon from non-*E*. *hirae* isolates was sequenced on an ABI Prism 3130xl genetic analyzer (Thermo Fisher Scientific Inc., Mississauga, ON, Canada) to differentiate *Enterococcus* spp. A two-tailed Fisher’s exact test was used in R v. 4.0.3 to compare the frequency of Enterococcus-positive samples by sample location for isolates from Enterococcosel agar with and without erythromycin. *P* values were corrected for multiple comparisons using the Benjamini-Hochberg method.

### Antimicrobial resistance screening of enterococci isolates.

Due to their well-documented use as indicator bacterial species, a random selection of isolates within each location and sample type and with a *groES*-*EL* spacer region that was 100% identical to E. faecalis or E. faecium were screened for ARGs and antimicrobial sensitivity. Broth microdilution with the Sensititre NARMS (National Antimicrobial Resistance Monitoring System) Gram-positive CMV3AGPF AST plate (Trek Diagnostics, Independence, OH, USA) was used to determine the susceptibility of 120 E. faecalis and 9 E. faecium isolates to 16 different antimicrobials. For antimicrobials in the panel, MIC breakpoints for *Enterococcus* spp. established by the Clinical and Laboratory Standards Institute (CLSI), European Committee on Antimicrobial Susceptibility Testing (EUCAST), or NARMS were used to interpret the results (Table S6). These isolates were also screened via PCR for the presence of the ARGs *erm*(B), *msrC*, *tet*(B), *tet*(C), *tet*(L), *tet*(M), *vanA*, *vanB*, and *vanC1* as described in Beukers et al. (2) (Table S7).

### Sequencing of selected Enterococcus faecalis and Enterococcus faecium isolates.

Forty-seven E. faecalis and eight E. faecium isolates were selected for whole-genome sequencing based on their AMR profiles and sample origin. Briefly, the isolates were recultured from the frozen glycerol on Enterococcosel agar and incubated for 24 h at 37°C to obtain isolated colonies with typical morphology and color. A single colony was then streaked onto BHI agar (Dalynn Biologicals) and grown overnight at 37°C, and colonies from this plate were suspended in 10 mM Tris-1mM EDTA (TE; pH 8.0) buffer to obtain an optical density at 600 nm (OD_600_) of 2.0 (2 × 10^9^ cells mL^−1^). One milliliter of this suspension was pelleted via centrifugation at 14,000 × *g* for 2 min. Genomic DNA was extracted from the pellet using the DNeasy blood and tissue kit (Qiagen, Mississauga, ON, Canada) with the modification that cells were incubated with agitation (150 rpm) for 45 min at 37°C in 280 μL of lysis buffer (20 mM Tris-HCl [pH 8.0], 2 mM sodium EDTA, 1.2% Triton X-100, and 20 mg mL^−1^ lysozyme) (Sigma-Aldrich Canada) prior to the addition of proteinase K and 5 μL of 100 mg mL^−1^ RNase A (Qiagen). The DNA concentration was determined using a Qubit fluorometer (Thermo Fisher Scientific, Mississauga, ON, Canada). The Nextera XT DNA library preparation kit (Illumina Inc., San Diego, CA, USA) was used to prepare sequencing libraries that were sequenced on a MiSeq instrument (Illumina Inc.) with the MiSeq reagent kit v3 (Illumina Inc.; 600 cycles) or on a NovaSeq 6000 machine (Illumina Inc.) with an SP flow cell (300 cycles).

### Genomic analysis of Enterococcus faecalis and Enterococcus faecium isolates.

Trimmomatic v. 0.39 (40) was used to remove sequencing adapters, reads with a quality score of less than 15 over a 4-bp sliding window, and reads that were less than 50 bp long. Genomes were assembled with SPAdes v. 3.15.1 (41) in “isolate mode,” and the quality of the assemblies was assessed with QUAST v. 5.0.2 (42). Potential contamination within each assembly was determined using Kraken 2 v. 2.1.1 and the minikraken2 database v. 2 (43) as well as CheckM v. 1.1.3 (44). GTDB-tk v. 1.3.0 (45) was also used to confirm the taxonomic assignments of the assemblies, and Prokka v. 1.14.6 (46) was used to annotate the assemblies. Determination of MLST was done on the assembled genomes using the E. faecalis (https://pubmlst.org/efaecalis) and E. faecium (https://pubmlst.org/efaecium/) MLST databases (47, 48).

The accessory, core, and pan-genome of the E. faecalis and E. faecium genomes were identified using Roary v. 3.13.0 (49) with a BLASTp identity cutoff of ≥95%. The core genome is defined as genes present in ≥99% of genomes. The core genes for both species were aligned in Roary using MAFFT v. 7.475 (50), and a maximum likelihood phylogenetic tree was inferred from this alignment using RAxML v. 8.2.12 (51) and viewed with ggtree v. 2.4.1 (52) in R. Several publicly available E. faecalis and E. faecium assemblies from various isolation sources, including humans and cattle in Alberta, were also included in the core and pan-genome analysis as listed in Table S8. The genome assemblies were also screened for virulence genes using the VirulenceFinder 2.0 database (53) and BLASTn (≥90% identity) and for ARGs using the CARD v. 3.0.9 (54) Resistance Gene Identifier (RGI). The depicted gene regions containing ARGs were constructed and validated using contig alignments in Geneious v. 11.0.9. BLAST was used to identify highly similar regions with >80% pairwise identity in bacterial strains present in NCBI.
