# Supplementary material for: Antimicrobial Resistance in Enterococcus Spp. Isolated from a Beef Processing Plant and Retail Ground Beef
Source: Microbiol Spectr. 2021 Nov 17;9(3):e01980-21. doi: 10.1128/Spectrum.01980-21 (PMC8597637; doi:10.1128/Spectrum.01980-21)
Supplement: SUPPLEMENTAL FILE 2 — Supplemental material. Download SPECTRUM01980-21_Supp_2_seq5.pdf, PDF file, 0.1 MB [file spectrum01980-21_supp_2_seq5.pdf]

Table S7. PCR primers used to amplify antimicrobial resistance genes in *Enterococcus faecalis* and *Enterococcus faecium* isolates.

Gene targeted	Sequence	Annealing temperature (°C)	Product size	REF
erm(B)	F-5'-GATACCGTTTACGAAATTGG-3'<br>R-5'-GAATCGAGACTTGAGTGTGC-3'	54	364	(1)
msrC	F-5′-TCGTTTTGTCATGAGACAAACAG-3′<br>R-5′-AAATTAGTCGGTTCATCTAACAG-3′	53	191	(2)
tet(B)	F-5'-ACACTCAGTATTCCAAGCCTTTG-3'<br>R-5'-GATAGACATCACTCCCTGTAATGC-3'	60	205	(3)
tet(C)	F-5'-CTTGAGAGCCTTCAACCCAG-3'<br>R-5'-ATGGTCGTCATCTACCTGCC-3'	55	418	(4)
tet(L)	F-5'-TCGTTAGCGTGCTGTCATTC-3'<br>R-5'-GTATCCCACCAATGTAGCCG-3'	55	267	(4)
tet(M)	F-5'-GTGGACAAAGGTACAACGAG-3'<br>R-5'-CGGTAAAGTTCGTCACACAC-3'	55	406	(4)
vanA	F-5'-TCTGCAATAGAGATAGCCGC-3'<br>R-5'-GGAGTAGCTATCCCAGCATT-3'	52	377	(5)
vanB	F-5'-CATCGCCGTCCCCGAATTTCAAA<br>R-5'-GATGCGGAAGATACCGTGGCT	50	298	(6)
vanC1	F-5'-GACCCGCTGAAATATGAAG<br>R-5'-CGGCTTGATAAAGATCGGG	50	420	(7)

1. Chen J, Yu Z, Michel FC, Jr., Wittum T, Morrison M. 2007. Development and application of real-time PCR assays for quantification of *erm* genes conferring resistance to macrolides-lincosamides-streptogramin B in livestock manure and manure management systems. Appl Environ Microbiol 73:4407-16. https://doi.org/:10.1128/AEM.02799-06.
2. Beukers AG, Zaheer R, Cook SR, Stanford K, Chaves AV, Ward MP, McAllister TA. 2015. Effect of in-feed administration and withdrawal of tylosin phosphate on antibiotic resistance in enterococci isolated from feedlot steers. Front Microbiol 6:483. https://doi.org/:10.3389/fmicb.2015.00483.

3. Peak N, Knapp CW, Yang RK, Hanfelt MM, Smith MS, Aga DS, Graham DW. 2007. Abundance of six tetracycline resistance genes in wastewater lagoons at cattle feedlots with different antibiotic use strategies. Environ Microbiol 9:143-151. https://doi.org/:10.1111/j.1462-2920.2006.01123.x.

4. Ng LK, Martin I, Alfa M, Mulvey M. 2001. Multiplex PCR for the detection of tetracycline resistant genes. Mol Cell Probes 15:209-15. https://doi.org/:10.1006/mcpr.2001.0363.

5. Klare I, Heier H, Claus H, Reissbrodt R, Witte W. 1995. *vanA*-mediated high-level glycopeptide resistance in *Enterococcus faecium* from animal husbandry. FEMS Microbiol Lett 125:165-71. https://doi.org/:10.1111/j.1574-6968.1995.tb07353.x.

6. Klare I, Konstabel C, Mueller-Bertling S, Werner G, Strommenger B, Kettlitz C, Borgmann S, Schulte B, Jonas D, Serr A, Fahr AM, Eigner U, Witte W. 2005. Spread of ampicillin/vancomycin-resistant Enterococcus faecium of the epidemic-virulent clonal complex-17 carrying the genes esp and hyl in German hospitals. Eur J Clin Microbiol Infect Dis 24:815-25. https://doi.org/:10.1007/s10096-005-0056-0.

7. Sahm DF, Free L, Handwerger S. 1995. Inducible and constitutive expression of vanC-1-encoded resistance to vancomycin in Enterococcus gallinarum. Antimicrob Agents Chemother 39:1480-4. https://doi.org/:10.1128/aac.39.7.1480.
